# Mechanism-Based Biomarker Prediction for Low-Grade Inflammation in Liver and Adipose Tissue

**DOI:** 10.3389/fphys.2021.703370

**Published:** 2021-11-10

**Authors:** Jolanda H. M. van Bilsen, Willem van den Brink, Anita M. van den Hoek, Remon Dulos, Martien P. M. Caspers, Robert Kleemann, Suzan Wopereis, Lars Verschuren

**Affiliations:** ^1^Department of Risk Assessment for Products in Development, The Netherlands Organization for Applied Scientific Research (TNO), Utrecht, Netherlands; ^2^Department of Microbiology and Systems Biology, The Netherlands Organization for Applied Scientific Research (TNO), Zeist, Netherlands; ^3^Department of Metabolic Health Research, The Netherlands Organization for Applied Scientific Research (TNO), Leiden, Netherlands

**Keywords:** mechanism, low-grade inflammation, blood-based biomarker, metabolic disease, lifestyle intervention

## Abstract

Metabolic disorders, such as obesity and type 2 diabetes have a large impact on global health, especially in industrialized countries. Tissue-specific chronic low-grade inflammation is a key contributor to complications in metabolic disorders. To support therapeutic approaches to these complications, it is crucial to gain a deeper understanding of the inflammatory dynamics and to monitor them on the individual level. To this end, blood-based biomarkers reflecting the tissue-specific inflammatory dynamics would be of great value. Here, we describe an *in silico* approach to select candidate biomarkers for tissue-specific inflammation by using *a priori* mechanistic knowledge from pathways and tissue-derived molecules. The workflow resulted in a list of candidate markers, in part consisting of literature confirmed biomarkers as well as a set of novel, more innovative biomarkers that reflect inflammation in the liver and adipose tissue. The first step of biomarker verification was on murine tissue gene-level by inducing hepatic inflammation and adipose tissue inflammation through a high-fat diet. Our data showed that *in silico* predicted hepatic markers had a strong correlation to hepatic inflammation in the absence of a relation to adipose tissue inflammation, while others had a strong correlation to adipose tissue inflammation in the absence of a relation to liver inflammation. Secondly, we evaluated the human translational value by performing a curation step in the literature using studies that describe the regulation of the markers in human, which identified 9 hepatic (such as Serum Amyloid A, Haptoglobin, and Interleukin 18 Binding Protein) and 2 adipose (Resistin and MMP-9) inflammatory biomarkers at the highest level of confirmation. Here, we identified and pre-clinically verified a set of *in silico* predicted biomarkers for liver and adipose tissue inflammation which can be of great value to study future development of therapeutic/lifestyle interventions to combat metabolic inflammatory complications.

## Introduction

Inflammation is an important component of normal responses to infection and injury, whether locally confined or systemic. An healthy immune response follows a characteristic pathway, where the first response is strong but short, resulting in the exclusion of the pathogen/damage followed by a recovery to homeostasis. This characteristic pathway of inflammation is essential for recovery and remodeling of tissues and helps to regain a healthy homeostasis including its critical function (Hotamisligil and Erbay, 2008). The evolutionary benefits of an optimal effective immune system are evident in protecting against pathogenic intruders. Since immune responses are also linked to energy metabolism, it can therefore be argued that the integration of these systems and their cooperation in responding to fluctuations in the energy and nutritional environment would be beneficial. These responses, however, need to be temporally and locally regulated to maintain an healthy homeostasis.

Chronic disruption of metabolic homeostasis that occurs in, for example, overnutrition, could lead to disturbed immune responses. Especially when the chronic inflammatory activation occurs in metabolically important organs such as liver and adipose tissue, these tissues are stimulated to produce pro-inflammatory cytokines, acute phase proteins, pro-inflammatory lipids, and other biological inflammatory mediators into the circulation, leading to a systemic inflammatory condition (Gregor and Hotamisligil, 2011; Minihane et al., 2015; Hotamisligil, 2017). These processes have a crucial role in the chronic metabolic disease development, such as obesity, type 2 diabetes, fatty liver disease and cardiovascular disease (Hotamisligil, 2006), and forms the mechanistic basis for risk factors of viral infections, such as coronavirus disease 2019 (COVID-19) (Stefan et al., 2021). In order to support therapeutic approaches of metabolic diseases, it is crucial to gain a deeper understanding regarding the inflammatory dynamics in time to monitor or even predict the homeostatic inflammatory status of the individual. To this end, tissue-derived plasma biomarkers reflecting the tissue-specific and systemic inflammatory dynamics in time would be of great help.

Several systemic pro-inflammatory markers such as C-reactive protein (CRP), interleukin (IL) 6, IL 18, fibrinogen, and adhesion molecules [e.g., E-selectin, intercellular adhesion molecule 1 (ICAM-1), and vascular cell adhesion protein 1 (VCAM-1)] (Pradhan, 2001; Thorand et al., 2005; Kelesidis et al., 2006; Li et al., 2009; Shibata et al., 2009) have been identified to monitor “end-stage” of chronic-low-grade inflammatory diseases such as type 2 diabetes, cardiovascular disease (CVD), and cancer. In contrast, plasma levels of the anti-inflammatory adiponectin were inversely associated with CVD (Shibata et al., 2009), type 2 diabetes (Li et al., 2009), and obesity-related cancer (Kelesidis et al., 2006). To know whether these and other biomarkers represent early stages of disease progression at the tissue level, and not at the systemic level, careful assessment of inflammatory biomarkers is required at the pathway level to select those markers that enable assessment of tissue-derived sub-clinical low-grade inflammation.

Recent advances in high-throughput technologies have made it possible to generate, analyse and integrate large multi-omics datasets at molecular and cellular levels (genes, proteins, metabolites, cells) to identify molecular markers of disease processes. The increasing use of *in silico* approaches and bioinformatics has encouraged researchers to use these multi-level datasets together with existing knowledge and databases in order to generate a systems-level overview of disease development (Mcdermott et al., 2013; Vafaee et al., 2018). GeneSet Enrichment Analysis (GSEA) has become the golden standard in the analysis of omics data, thereby reducing the complexity of the analyses and providing a systems view of the biological processes involved in disease development (Khatri et al., 2012). A large number of methods have been proposed in the literature for this task. The majority of these methods use expression levels as input together with their associated biological pathways (Khatri et al., 2012; García-Campos et al., 2015). The approach described here inverts this strategy, taking known pathways and tissue-derived biomolecules *a priori*, thereby creating immediately interpretable candidate biomarkers that may help the monitoring of disease at an early phase and/or support treatment strategies. Focusing on the key events leading to chronic low-grade inflammation, enabled us to focus on generic features of a broad range of metabolic diseases. We developed an *in silico*-based approach which uses prior knowledge on dysregulated pathways in chronic low-grade inflammation to predict blood-based candidate biomarkers. We verified this approach in biomarker databases, experimental data, and scientific literature to identify blood-based biomarkers reflecting the dynamic inflammatory status during the sub-clinical process of chronic low-grade inflammation in liver and adipose tissue.

## Materials and Methods

### Selection of Genes From Gene Ontology and Human Protein Atlas

**FIGURE 1:**
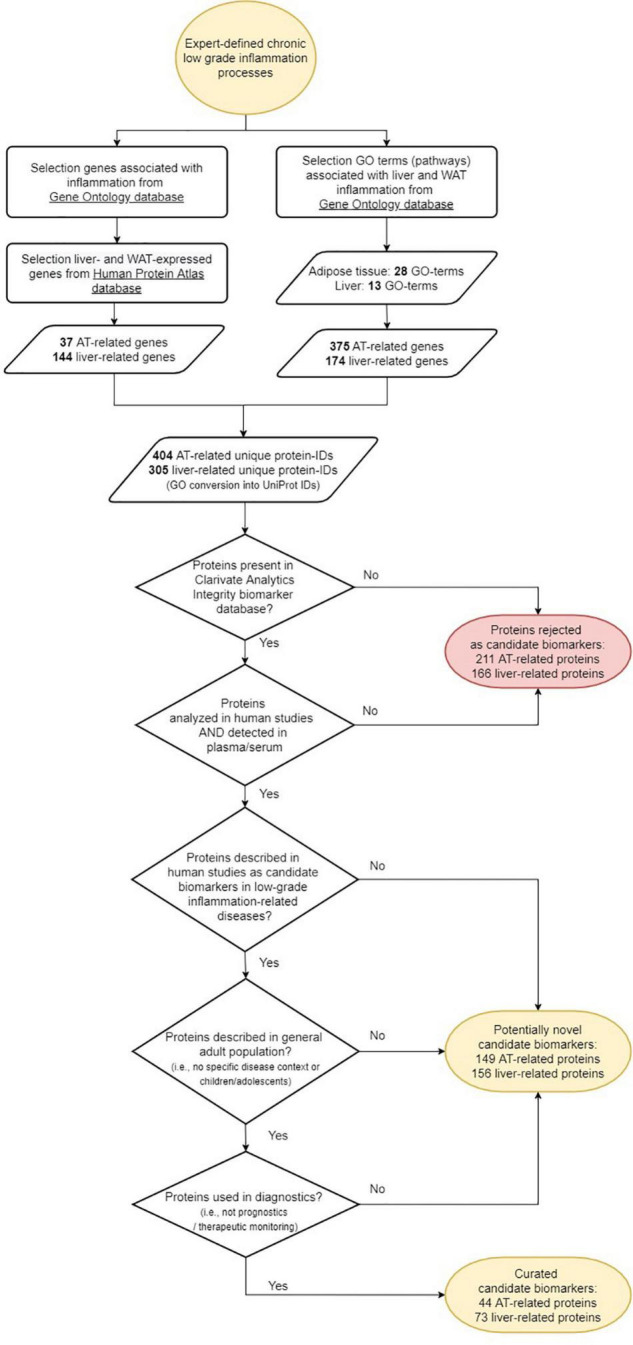
Workflow of the selection of candidate protein biomarkers for tissue inflammation of liver and/or adipose tissue.

The workflow of the biomarker selection approach used in this study is visualized in Figure 1. Briefly, an initial selection of gene ontology (GO) terms was listed using the search terms ‘‘adipose’’ or ‘‘adipocyte’’ to identify adipose tissue related GO terms, and ‘‘liver,’’ ‘‘hepatic,’’ or ‘‘hepatocyte’’ to identify liver tissue related GO terms in the Gene Ontology knowledgebase (^1^ accessed September 2020). The list of GO terms was manually curated and only those that were associated with human, non-embryonic, endogenous and tissue-specific processes were selected, together with their “child” terms (i.e., a more specialized term than their broader “paren” term). Subsequently, all immune related genes from the GO knowledgebase were selected from the GO terms “immune system process,” “inflammatory response,” “cytokine production,” and their child terms. In order to select those tissue-specific pathways potentially associated with inflammation, only those adipose and liver GO terms were taken that contained genes also present in immune-related GO terms (Meijerink et al., 2019). The genes from these adipose and liver GO terms were then extracted from the GO knowledgebase under the conditions of species “homo sapiens” and type “protein.”

Parallel to the extraction of genes from the GO knowledgebase, tissue-specific genes for adipose tissue and liver were selected from the Human Protein Atlas (HPA) (^2^ accessed September 2020). Details on the definition of tissue specificity can be found in the HPA documentation. In short, it is defined as at least four-fold higher mRNA level in a particular tissue or a group of 2–5 tissues compared to any other tissue or the average level in all other tissues. Only immune related genes as defined from the GO knowledgebase described above and those genes that were assigned a UniProt ID or a gene name were selected.

### Selection and Curation of Candidate Protein Biomarkers

All gene names and the associated UniProt ID’s from the selected genes were compared to biomarkers from the Clarivate Analytics Integrity biomarker database^3^ labeled with type “proteome,” substrate “plasma” or “serum,” and evidence “early human studies,” “late human studies,” or “recommended/approved” to select only those candidate markers that are being identified as human protein biomarker in blood.

Thereafter, the candidate biomarker set was assessed for its novelty using 3 criteria: (i) candidate biomarker is described in one of the following low-grade inflammation related diseases or conditions (Clarivate Analytics Integrity term: “condition”): metabolic syndrome, type 2 diabetes, hyperglycemia, prediabetes, insulin resistance, glucose intolerance, dyslipidemia, hyperlipidemia, hypertriglyceridemia, or atherosclerosis; (ii) candidate biomarker is commonly analyzed in the human adult population; (iii) candidate biomarker is used as diagnostic biomarker in the diseases or conditions under the first criterium. If the answer was no to one of these 3 criteria, the candidate biomarker was assigned as potentially “novel” candidate biomarker. If the answer was yes to these 3 criteria, the candidate biomarker was assigned “curated” candidate biomarker.

### Verification of Candidate Biomarkers

#### Obese Ldlr−/−.Leiden Mice

The *in silico* predicted candidate biomarkers were verified for their suitability as markers for liver- and/or adipose tissue inflammation in male 14–16 week-old obese and inflammation-prone Ldlr−/−.Leiden mice (van den Hoek et al., 2020). Animal care and use were performed in accordance with the general principles governing the use of animals in experiments of the European Communities (Directive 86/609/EEC) and Dutch Legislation (The Experiments on Animals Act, 1997). This included approval of the study by the ethical review committee (approval reference number TNO-312). The control and reference group of a larger study evaluating the organ-specific effects of lifestyle interventions (van den Hoek et al., 2021) were used for biomarker verification. Briefly, two experimental groups of Ldlr−/−.Leiden mice were included in the study: one group of mice (*n* = 10; healthy reference group) were fed a low-fat grain-based chow diet (R/M-H, Ssniff Spezialdieten GmbH, Soest, Germany) and the second group of mice (*n* = 17) were fed a high fat diet (HFD) containing 45 kcal% fat from lard, 35 kcal% from carbohydrates and 20 kcal% casein (D12451, Research Diets, new Brunswick, NJ, United States) for 50 weeks to induce an obese phenotype. Mice were group housed in a temperature-controlled room on a 12 h light-dark cycle and had free access to food and heat sterilized water. Body weight and food intake were determined regularly during the study. At *t* = 50 weeks mice were sacrificed unfasted using gradual-fill CO_2_ asphyxiation. Liver tissue and perigonadal white adipose tissue (WAT) were collected, weighed and fixed in formalin and paraffin-embedded for histological analysis or fresh-frozen in N_2_ and subsequently stored at -80°C for gene expression analysis. Hepatic inflammation was scored using 3 μm sections which were stained with hematoxylin and eosin (H&E) and by counting the number of aggregates of inflammatory cells per field using a 100x magnification (view size of 4.2 mm^2^). The averages of five random non-overlapping fields were taken and values were expressed per mm^2^. Perigonadal WAT inflammation was scored using 5 μm sections which were stained with hematoxylin-phloxine-saffron (HPS) using 3 randomly selected fields (1.56 mm^2^ for perigonadal WAT). Adipose tissue inflammation was measured by counting crown-like structures (CLS) per field using a 100× magnification and values were expressed as number of CLS per 1000 adipocytes. Plasma levels of Serum Amyloid A (SAA) were measured by ELISA specific for SAA (Invitrogen, # KMA0021).

#### Transcriptome Analysis of Liver and Adipose Tissue

To perform gene expression analysis RNA isolation was performed as described previously in detail (Verschuren et al., 2014). Total RNA was extracted from fresh frozen liver and perigonadal WAT samples using glass beads and RNA-Bee (Campro Scientific, Veenendaal, Netherlands). Briefly, mRNA was extracted from total RNA using oligo-dT magnetic beads. After fragmentation of the mRNA, cDNA was synthesized followed by ligation with the sequencing adapters and amplified by PCR. Quality and yield of the amplicon was determined (Fragment Analyzer, Agilent Technologies, Amstelveen, Netherlands) and fulfilled QC-criteria (broad peak between 300 and 500 bp). In total, a concentration of 1.1 nM of amplicon-library DNA was used for sequencing. Clustering and DNA sequencing, using the Illumina NovaSeq6000, was performed according to manufacturer’s protocols by service provider GenomeScan B.V (Leiden, Netherlands), yielding 15–40 million sequencing clusters per sample and 2 × 150 bp Paired-End reads (PE) per cluster. These counts serve as input for the statistical analysis using DEseq2 package (Love et al., 2014). Selected differentially expressed genes (DEGs), corrected for multiple testing (available in DEseq2 package), were used for expression analyses.

#### Verification in Human Studies

The candidate biomarkers for low grade inflammation in liver and/or WAT that were verified in the obese Ldlr−/−.Leiden mice, were further evaluated for their confirmed use as hepatic or adipose inflammation markers in human in the MEDLINE^®^ database. The database was searched during February 2021 and March 2021 using the biomarker names and the tissue names. Publications were manually screened and evaluated to assess the use of the candidate biomarkers in a human inflamed setting. The level of confirmation was determined based on (i) a mechanistic rationale present for the marker to be related to liver or adipose tissue; (ii) the biomarker has been described to be associated with tissue specific diseases (e.g., steatosis for liver or obesity for adipose tissue); (iii) the biomarker has been described to be associated with one or more metabolic disorders (i.e., metabolic syndrome, type 2 diabetes, hyperglycemia, prediabetes, insulin resistance, glucose intolerance, dyslipidemia, hyperlipidemia, hypertriglyceridemia, or atherosclerosis).

Three levels of biomarker confirmation were determined: The biomarker was regarded confirmed (level 3) as tissue inflammation derived biomarker if all three conditions were met; putative (level 2) if two out of three conditions were met; possible (level 1) if only one condition was met, being either condition i or ii; not confirmed (level 0) if none of the conditions or only condition 3 was met.

### Statistics

All values shown in graphs represent means ± Standard Error of the Mean (SEM). Statistical differences between groups were determined by using non-parametric Kruskal-Wallis followed by Mann-Whitney *U* test for independent samples using SPSS software. Two-tailed p-values were used and P-values < 0.05 was considered statistically significant. In the case of transcriptome analysis, differentially expressed genes were selected using p-values adjusted for multiple testing (available in DEseq2 package; False Discovery Rate, FDR) < 0.01. Candidate biomarkers were selected based the following criteria: (a) biomarker is detected on gene level in tissue from the mouse study; (b) biomarker is significantly different in liver or adipose tissue; (c) expression (count) level in target tissue is higher than expression in non-target tissue. Spearman’s Rank correlation analysis (MS office package, MS-Excel) was performed to calculate the relation between the absolute gene expression in the tissue and the quantified inflammatory aggregates per animal. A correlation cut-off value of ±0.6 was considered relevant for further evaluation.

## Results

### Selection of Genes From Gene Ontology and Human Protein Atlas

Table 1 presents an overview of human gene ontologies and genes that were selected as input for the biomarker workflow (Figure 1). For adipose tissue, the initial database search retrieved 47 gene ontologies of which 9 parent ontologies were manually selected (Supplementary Table 1). After extension with their child terms, 41 ontologies were selected for adipose tissue. After mapping with immune-related genes, 7 parent adipose tissue ontologies with 28 child ontologies were selected for further processing (Table 1). These ontologies contained 43% (20%–100%) genes that were also present in immune related ontologies. The search for liver ontologies resulted in 115 hits of which 15 ontologies were selected (Supplementary Table 2). With their child ontologies, a total of 37 terms were selected for liver. A number of 8 parent liver ontologies with their 13 child ontologies were selected for further processing based on their overlap with immune ontologies (Table 1). In these ontologies, 45% (25%–100%) of the genes were also related to immune GO terms. Overall, 375 adipose tissue related genes and 174 liver associated genes were selected from the GO knowledgebase.

**TABLE 1:** Selected parent gene ontologies with the number of child ontologies and genes, as well as the number genes also present in immune related ontologies.

**Tissue**	**GO accession**	**GO term (parent)**	**Number of child ontologies**	**Number of genes**	**Number of immune genes (%)**
Adipose tissue	GO:0070162	adiponectin secretion	2	6	3(50%)
	GO:0060612	adipose tissue development	4	38	19(50%)
	GO:0005901	Caveola	5	83	30(36%)
	GO:1904606	fat cell apoptotic process	1	1	1(100%)
	GO:0045444	fat cell differentiation	9	213	94(44%)
	GO:0070341	fat cell proliferation	2	10	2(20%)
	GO:0044321	response to leptin	5	24	11(46%)
	Additional from Human protein atlas			69	29(42%)

Liver tissue	GO:0034382	chylomicron remnant clearance	1	8	3(38%)
	GO:0002384	hepatic immune response	1	2	2(100%)
	GO:0035733	hepatic stellate cell activation	2	6	3(50%)
	GO:0061868	hepatic stellate cell migration	1	2	1(50%)
	GO:1990922	hepatic stellate cell proliferation	1	2	1(50%)
	GO:0097284	hepatocyte apoptotic process	1	12	6(50%)
	GO:0001889	liver development	5	130	60(46%)
	GO:0034379	very-low-density lipoprotein particle assembly	1	12	3(25%)
	Additional from Human protein atlas			326	131(40%)

Additionally, a number of 79 adipose and 359 liver specific genes were retrieved from the Human Protein Atlas. Of these genes, 29 adipose and 131 liver specific immune-associated genes were a unique addition to the genes selected from GO.

### Selection of Candidate Protein Biomarkers

Of the 404 adipose related genes selected from the Gene Ontology database and Human Protein Atlas database, 44 genes qualified as a curated candidate plasma protein biomarker of adipose tissue inflammation (Figure 1 and Table 2). Adipose processes that were highly represented included caveola (ADCY8, SELE, INSR, IRS1, TFPI, HMOX1, FASLG, NOS3), fat cell differentiation (SREBF1, METRNL, GPX1, RETN, TNF, FRZB, LPL, RARRES2, PPARG, ADIPOQ, SORT1, TGFB1, IL6), brown fat cell differentiation (ADIPOQ, FABP4, METRNL, FABP3, LEP, FTO, METRNL, FNDC5), white fat cell differentiation (FABP4, PPARG), and response to leptin (LEP, LEPR, FGF23, EDN1). Of the candidate biomarker list for adipose tissue LEP, ADIPOQ, LPL, FAB4, IL6, and PPARG are represented in the Human Protein Atlas as adipose tissue specific genes.

**TABLE 2:** The selected candidate biomarkers in each parent Gene Ontology term.

**Tissue**	**GO accession**	**GO term (parent)**	**Number of candidates for tissue specific inflammation^a^**	**names literature-confirmed biomarkers “candidate biomarkers confirmed in biological context”**
Adipose tissue	GO:0070162	adiponectin secretion	1	IL1B^#^
	GO:0060612	adipose tissue development	6	NAMPT^#^; SORL1^#^; **LEP**^#^; FTO; PIK3CA^#^; GHRL^#^
	GO:0005901	Caveola	8	ADCY8^#^; SELE^#^; INSR; IRS1; TFPI; HMOX1^#^; FASLG^#^; NOS3
	GO:1904606	fat cell apoptotic process	1	**LEP** ^#^
	GO:0045444	fat cell differentiation	18	**LEP**^#^; FTO; FRZB; **ADIPOQ**^#^; SORT1; SREBF1; **LPL**^#^; RARRES2^#^; **FABP4**^#^; TGFB1^#^; TNF^#^; METRNL^#^; GPX1^#^; FABP3; RETN^#^; FNDC5; **IL6**^#^; **PPARG**^#^
	GO:0070341	fat cell proliferation	1	FTO
	GO:0044321	response to leptin	4	**LEP**^#^; LEPR^#^; FGF23; EDN1^#^
	Additional from Human protein atlas		10	**ACP5**^#^**;CD36**^#^**;CHIT1**^#^**;GPNMB**^#^**;ITLN1**^#^**;MMP9**^#^**;PLA2G7**^#^**;** **PRG4**^#^**;PTX3**^#^**;SAA1**^#^

Liver tissue	GO:0034382	chylomicron remnant clearance	7	LDLR^#^; LIPC; **APOB**^#^; **APOC3**; **APOC2**; **APOC1**; **APOE**^#^
	GO:0002384	hepatic immune response	2	IL6R^#^; IL6^#^
	GO:0035733	hepatic stellate cell activation	2	LEP^#^; **GCLC**
	GO:0061868	hepatic stellate cell migration	0	
	GO:1990922	hepatic stellate cell proliferation	0	
	GO:0097284	hepatocyte apoptotic process	2	GSN^#^; KRT18
	GO:0001889	liver development	11	**FGL1**^#^; **HAMP**^#^; **CPB2**^#^; EGFR^#^; PIK3CA^#^; REG1A^#^; HMOX1^#^; **VTN**^#^; IL10^#^; **PCSK9**; FGF1
	GO:0034379	very-low-density lipoprotein particle assembly	3	**APOB**^#^; **APOC3**; **APOC1**
	Additional from Human protein atlas		49	**A1BG**^#^**;A2M**^#^**; ADAMTS13**^#^**;AGT**^#^**;AHSG**^#^**;AMBP**^#^**;APOA1**^#^**;** **AZGP1**^#^**; CAT**^#^**;CD14**^#^**;CHI3L1**^#^**;CLU**^#^**;AGTR1**^#^**;CPN2**^#^**;F7**^#^**;** **FST**^#^**;GDF2**^#^**;HP**^#^**;HRG**^#^**;ICAM1**^#^**;IGFBP2**^#^**;IL18BP**^#^**;IL27**^#^**;** **KLKB1**^#^**;KNG1**^#^**;LBP**^#^**;LEPR**^#^**;LRG1**^#^**;MASP1**^#^**;MBL2**^#^**;ORM1**^#^**;** **PLA2G2A**^#^**;PLG**^#^**;PRDX4**^#^**;PRG4**^#^**;PROC**^#^**;PROS1**^#^**; RARRES2**^#^**;** **RBP4**^#^**;SAA1**^#^**;SDC1**^#^**;SERPINA1**^#^**;SERPINA3**^#^**;SERPINC1**^#^**;** **SOD1**^#^**;TF**^#^**; SERPING1**^#^**; TNFSF14**^#^**;TTR**^#^

*Candidate biomarkers that represent the overlap with immune GO terms are indicated with an #. Candidate biomarkers identified as tissue-specific marker according to the Human Protein Atlas database are bold-marked and underlined. As some candidate biomarkers are present in multiple GO terms, the sum of these numbers per tissue exceeds the total number of selected genes presented in the text (44 for adipose tissue and 73 for liver tissue).*

Of the 305 liver-related genes selected from the Gene Ontology and Human Protein Atlas databases, a number of 73 genes qualified as a curated candidate plasma protein biomarker for hepatic tissue inflammation (Figure 1 and Table 2). Highly manifest liver processes included chylomicron remnant clearance (LIPC, APOB, APOC3, APOC2, APOC1, APOE), liver regeneration (HAMP, CPB2, EGFR, REG1A, HMOX1, VTN, IL10), and very-low-density lipoprotein particle assembly (APOB, APOC3, APOC1). In the case of the candidate biomarkers, APOB, APOC3, APOC2, APOC1, APOE, GCLC, FGL1, HAMP, CPB2, VTN, PCSK9, APOB, APOC3, APOC1, were also present in Human Protein Atlas as tissue specific genes.

### Verification in Obese Ldlr−/−.Leiden Mice

**FIGURE 2:**
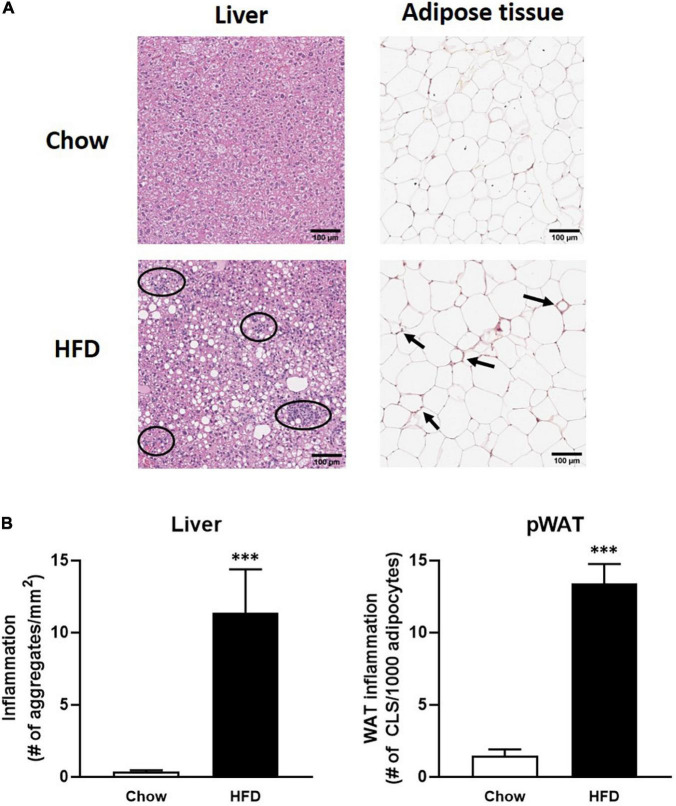
**(A)** Panels of immuno-histochemical staining of inflammatory aggregates in liver and adipose tissue; **(B)** quantification of inflammatory aggregates *in* HFD-fed mice (black bars) in liver (left) and adipose tissue (right) as compared to chow fed mice (open bars). ^∗∗∗^ indicates significance with *P*-values < 0.05.

The first step of biomarker verification is based on their expression in tissue upon increased inflammatory conditions. To this end, curated candidate biomarkers (Table 2) for inflammation in liver and adipose tissue were determined in liver and adipose tissue after 50 weeks of HFD treatment and compared to the expression under healthy (chow-fed) conditions. As compared to their chow-fed counterparts, HFD feeding resulted in a strong induced hepatic inflammation, characterized by inflammatory cell aggregates of immune cells (Figure 2A). Quantification of the number of inflammatory aggregates showed that the HFD feeding resulted in a strong increase in the number of aggregates as compared to chow fed animal (31.6-fold increase, *P* < 0.01). High fat diet feeding also resulted in increased inflammation in perigonadal WAT depot as compared to the chow fed controls (Figure 2B). Quantification of the data shows that HFD feeding increased the number of crown-like structures (CLS)/mm^2^ perigonadal WAT (9.1-fold, *p* < 0.001). In all, these data show an increased inflammatory status in both liver and adipose tissue of mice fed a HFD as compared to chow fed controls.

**FIGURE 3:**
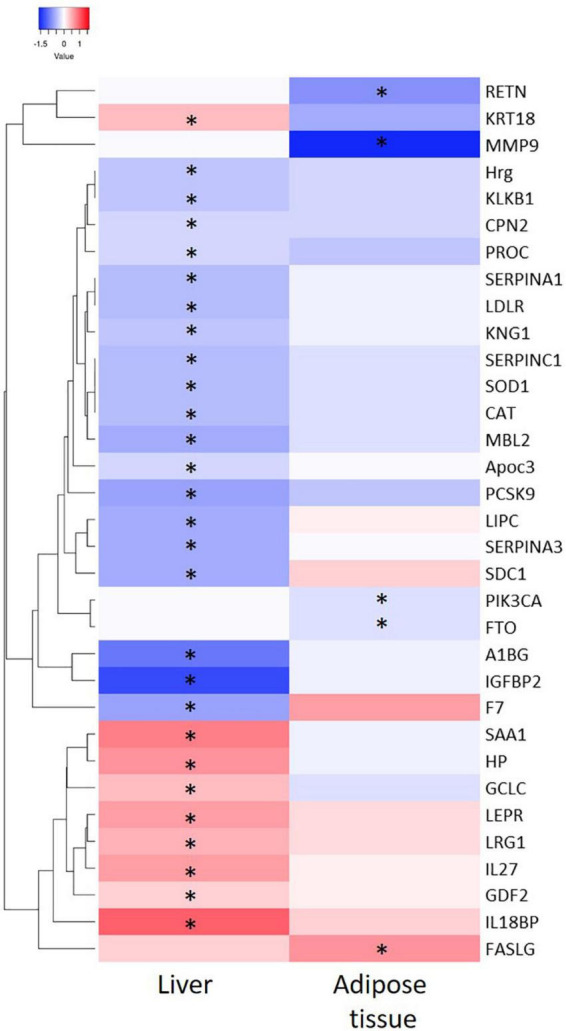
Heatmap representation of significant differential expressed genes from liver and adipose tissue and after 50 weeks of High Fat Diet feeding as compared to chow fed mice. ^∗^indicate significance of gene expression in specific tissue (*P* < 0.05).

Thereafter, the genes coding for the 44 adipose tissue-derived biomarker candidates and 73 liver tissue-derived biomarker candidates were analyzed in the inflamed tissues using RNAseq technology. A heatmap visualization of a selection of the candidate biomarker genes (see criteria in Materials and Methods section) is depicted in Figure 3. In inflamed livers from obese mice fed a HFD, the candidate biomarkers KRT18, SAA1, HP, GCLC, LEPR, LG1, IL27, GDF2, and IL18BP were significantly upregulated, whereas HRG, KLKB1, CPN2, PROC, SERPINA1, LDLR, KNG1, SERPINC1, SOD1, CAT, MBL2, APOC3, PCSK9, LIPC, SERPINA2, SDC1, A1BG, IGFBP2 and F7 are significantly downregulated in the inflamed livers. In the WAT from obese mice fed a HFD, FASLG expression was increased, while RETN, MMP9, PIK3CA, and FTO were significantly downregulated in the inflamed WAT.

Next step was to evaluate whether there is a relation between the differentially expressed biomarkers and the inflammatory state in liver and adipose tissue on the individual level. To this end we performed Spearman’s rank correlation to calculate the relation between the absolute gene expression in the tissue and the quantified inflammatory aggregates per animal (Table 3). This analysis shows a positive correlation of IL18BP, KRT18, SERPINA3, LRG1, LEPR, HP, and SAA1 with the level of inflammation in the liver and a negative correlation of IGFBP2, LIPC, MBL2, PCSK9, SERPINA1, SERPINC1, F7, LDLR, and CAT with the level of inflammation in the liver. In adipose tissue, a negative correlation of MMP9 and RETN expression was found with the level of inflammation.

**TABLE 3:** Spearman’s Rank correlation between the amount of inflammation in the tissue (liver and adipose tissue) and the expression of the candidate marker based on gene expression.

**Biomarker (gene/protein)**	**Correlation to hepatic inflammation**	**Correlation to adipose tissue inflammation**	**Literature confirmation as tissue-specific diagnostic biomarker**
MMP9/Matrix metalloproteinase 9	#*	−0.76	***Level 3 confirmation^a^.*** Matrix metalloproteinases (MMPs) have multiple functions, including tissue remodeling in response to injury. MMP-9 is mainly expressed in lymphoid tissue, blood and adipose tissue and involved in several biological processes, including inflammation (Nagase et al., 2006). Circulating levels of MMP-9 are increased with obesity, metabolic syndrome (MetS) and cardiovascular disease (Hopps et al., 2016; Jaoude and Koh, 2016; Ritter et al., 2017).
RETN/Resistin	#	−0.70	***Level 3 confirmation*** Resistin is an adipokine that is secreted by adipose tissue and stimulating expression of pro-inflammatory cytokines. Although there is some discussion, there is increasing consensus on the positive association between resistin and obesity, insulin resistance, and cardiovascular disease (Su et al., 2019; Recinella et al., 2020).
CAT/Catalase	–0.69	#	***Level 1 confirmation.*** Catalase is an antioxidant enzyme in microsomes of liver and plays a major role in detoxification of peroxides and reactive oxygen species (ROS) (Glorieux and Calderon, 2017). Overexpression of catalase protects against inflammation associated injury including atherosclerosis and diabetic complications (Góth, 2001)
F7/Coagulation Factor VII	–0.68	#	***Level 1 confirmation.*** Coagulation factor VII is synthesized in liver and adipose tissue and is part of the coagulation pathway where it binds to Tissue Factor (TF). Plasma concentrations of F7 were shown to be significantly higher in obese as compared to non-obese subjects (Lorenzet et al., 2012).
HP/Haptoglobin	0.85	#	***Level 3 confirmation.*** Haptoglobin is an acute phase protein, synthesized by the liver and scavenges hemoglobine in the circulation. Haptoglobin concentrations in the circulation are in healthy subjects very low, but in response to inflammation it is released rapidly in the circulation (Quaye, 2008).
IGFBP2/Insulin Like Growth Factor Binding Protein 2	–0.65	#	***Level 2 confirmation***. IGFBP2 protein levels are altered in type 2 diabetes mellitus patients and associated with cardiovascular disease risk factors. IGFBP2 concentration is lower in T2D patients vs healthy controls; inversely associated with pulse wave velocity in T2D and healthy controls (Hjortebjerg et al., 2017)
IL18BP/Interleukin 18 binding protein	0.75	#	***Level 3 confirmation.*** Human IL-18BP is secreted mostly by hepatocytes and macrophages in the liver. The binding protein binds the pro-inflammatory IL-18 and to a lesser extent also the anti-inflammatory IL-37 cytokine. Inherited IL-18BP deficiency underlies hepatitis by unleashing IL-18 (Belkaya et al., 2019). The IL-18/IL-18BP balance plays a role in several metabolic disorders, such as obesity, diabetes, and atherosclerosis, however, the exact mechanism is yet elusive (Kaplanski, 2018).
KRT18/Keratin 18	0.79	#	***Level 2 confirmation.*** Keratin 18 is expressed in epithelial tissue. Circulating levels of keratin 18 predict drug induced liver injury (Llewellyn et al., 2021), act as diagnostic and prognostic biomarker for acute alcoholic hepatitis (Vatsalya et al., 2020), and are associated with hepatic steatosis in elderly T2D patients (Morling et al., 2014). Keratin 18 had weak positive associations with several metabolic risk factors (glucose, HbA1c, BMI, waist, triglycerides) in elderly T2D patients (Morling et al., 2014).
LDLR/Low Density Lipoprotein Receptor	–0.64	#	***Level 3 confirmation.*** LDLR most investigated role is in the clearance of atherogenic Low Density Lipoproteins particles. However, LDLR can also be detected in circulation after cleavage by ADAM-17, an enzyme activated by inflammation (Mbikay et al., 2020).
LEPR/Leptin receptor	0.64	#	***Level 3 confirmation***. The action of leptin is mediated by the leptin receptor, a membrane bound receptor which can be cleaved from the membrane and detected in circulation (van Dielen et al., 2002). Leptin and soluble leptin receptor are independently and inversely associated with gestational diabetes (Mosavat et al., 2018). Soluble leptin levels are associated with pancreatic beta-cell dysfunction in T2DM patients (Morioka et al., 2018). Hepatic steatosis is negatively correlated with soluble Leptin receptor (Cernea et al., 2018).
LIPC/Hepatic Lipase	–0.65	#	***Level 1 confirmation.*** Hepatic lipase (HL) is an enzyme that hydrolysis triglycerides and has a putative role in the catabolism of HDL particles (Connelly, 1999). HL can either remain attached to the liver or is in free form in blood. HL deficiency causes hepatic inflammation in mice (Andrés-Blasco et al., 2015), the potential effect in humans needs to be studied.
LRG1/Leucine Rich Alpha-2-Glycoprotein 1	0.63	#	***Level 2 confirmation.*** LRG1 is highly abundant in the liver and has been associated with acute-phase response, being induced by pro-inflammatory cytokines (Pek et al., 2018). LRG1 was shown to be increased in T2D patients with vascular disease (Liu et al., 2020).
MBL2/Mannose Binding Lectin 2	–0.67	#	***Level 3 confirmation.*** Mannose-binding lectin 2 (MBL2) is an important component of the innate, non-specific immunity, primarily produced by liver (Gadjeva et al., 2004). MBL2 deficiency is known to affect defense against infections (Thiel et al., 2006). Serum MBL levels are significantly elevated in patients with type 1 diabetes (Hansen et al., 2003), and elevated serum levels of MBL can indicate poor diabetic control in T2DM patients (Elawa et al., 2011).
PCSK9/Proprotein convertase subtilisin/kexin type 9	–0.83	#	***Level 1 confirmation.*** Proprotein convertase subtilisin/kexin type 9 (PCSK9) is produced by the liver. In morbidly obese patients support a role for PCSK9 in liver fat accumulation, but no link was found for hepatic inflammation (Emma et al., 2020).
SAA1/Serum Amyloid A1	0.82	#	***Level 3 confirmation.*** Serum Amyloid A1 is an acute phase protein produced by hepatocytes in response to inflammation (Sack, 2018). SAA plasma concentrations in healthy subjects are normally very low, but in response to inflammation it is released rapidly in the circulation. SAA1 binds to HDL particles and therefore also play a role in atherosclerosis.
SERPINA1/Alpha 1-antitrypsin	–0.63	#	***Level 3 confirmation.*** α1-antitrypsin (A1AT) is an inhibitor of the pro-inflammatory protein neutrophil elastase. A1AT is produced in the liver and plasma levels are increased upon inflammatory response (Berger et al., 2018). A1AT deficiency is described to be associated with an increased risk of type 2 diabetes mellitus development (Sandström et al., 2008).
SERPINA3/Alpha-1-anti chymotrypsin	0.77	#	***Level 3 confirmation.*** Alpha-1-antichymotrypsin (AACT) is an acute phase protein synthesized in liver and induced during inflammation. Plasma levels of AACT are reduced with NASH (Hou et al., 2020) and with prediabetes, type 2 diabetes and abdominal obesity compared to normal (Kim et al., 2019).
SERPINC1/Anti-thrombin	–0.64	#	***Level 3 confirmation.*** Antithrombin is an anticoagulant glycoprotein with anti-inflammatory activities, produced by the liver (Ishikawa et al., 2017). Diabetic patients showed increased antithrombin-III compared with their non-diabetic counterparts (Murri et al., 2013).

* #: correlation coefficients in the range of-0.60 to 0.60. ^*a*^levels of confirmation of being a biomarker of tissue-specific inflammation are 0 (no confirmation), 1 (possible), 2 (putative) and 3 (confirmed).

**FIGURE 4:**
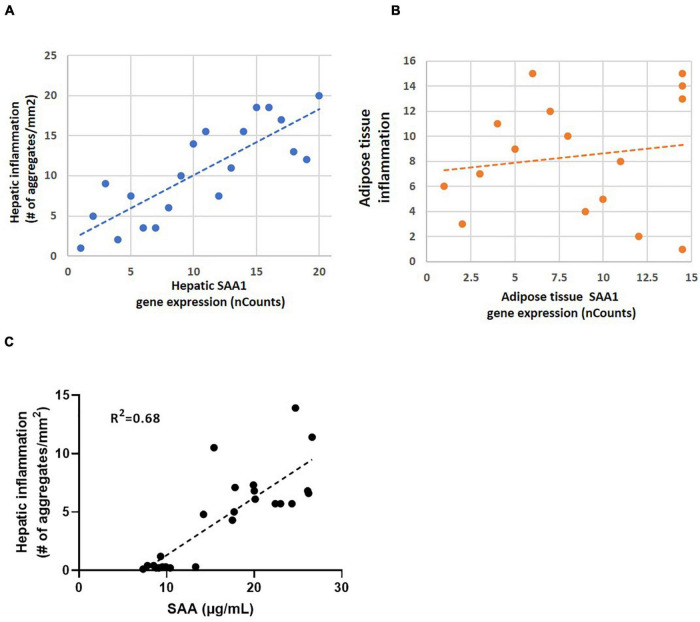
**(A)** Correlation analysis of hepatic inflammation based on immuno-histochemical staining of inflammatory aggregates in liver and hepatic SAA1 expression in mice (*R*^2^ = 0.82; *P* < 0.01); **(B)** Correlation analysis of adipose tissue inflammation based on immuno-histochemical staining of crown-like structures and adipose tissue SAA1 expression in mice (*R*^2^ = 0.15; *P* > 0.5); **(C)** Correlation analysis of hepatic inflammation in liver and plasma SAA1 levels (*R*^2^ = 0.68; *P* < 0.01).

To illustrate how a strong correlation between gene expression and inflammation of a specific gene can be translated in the concentration of a plasma marker SAA was analyzed. The experimental liver data shows a strong correlation between hepatic SAA1 expression and the number of hepatic inflammatory aggregates (Figure 4A) while a relation between adipose tissue gene expression and inflammation based on number of crown-like structures is clearly absent (Figure 4B). Biomarker analysis in plasma showed a good relation between hepatic inflammation and the concentration of plasma SAA (Figure 4C).

### Verification in Human Studies

The expression of 18 candidate biomarkers that correlated to inflammation in mice (Table 2, first three columns) could be further verified in literature as tissue-derived biomarker for adipose tissue and liver inflammation in humans (Table 3, last column). All 18 human candidate biomarkers could be more or less confirmed by literature: 4 out of 18 showed level 1 confirmation, 3 out of 18 showed level 2 confirmation, and 11 out of 18 showed level 3 confirmation.

As depicted in Table 3, the confirmed candidate biomarkers for inflammation in adipose tissue are linked to extracellular matrix processes (MMP-9) and to lipid metabolism (Resistin). The confirmed candidate liver inflammation biomarkers are linked to biological processes such as growth factors regulation (IGFBP2), serine proteases and inhibitors (coagulation factor VII, SERPINA1, SERPINA3, and SERPINC1), lipid metabolism (LEPR, LDLR, LIPC, and PCSK9), oxidative stress (Catalase), and immune response (SAA1, Haptoglobin, LRG1, MBL2, Keratin 18, and IL18BP). These data show that the *in silico* selection procedure, followed by *in vivo* verification in mice, results in a useful set of candidate biomarkers for human application.

## Discussion

Chronic inflammatory diseases are recognized as the leading cause of death in industrialized countries today, with the majority of deaths accountable to inflammation-related diseases such as cardiovascular disease, diabetes mellitus, non-alcoholic fatty liver disease (NAFLD) and autoimmune and neurodegenerative conditions (Roth et al., 2018). Research has shown that obesity, similar to the most chronic diseases, is characterized by an inflammatory state reflected by elevated circulating levels of pro-inflammatory proteins (de Heredia et al., 2012). Both the local and systemic responses initiated by an inflammatory process can result in a dysbalance in metabolism in the tissues affected referred to as chronic low-grade inflammation. It is important to explore the general molecular mechanisms that integrate the immune response with systemic and local metabolic homeostasis and to identify biomarkers that reflect these pathways for early diagnosis and prevention/treatment of chronic metabolic diseases. Organ-specific candidate biomarkers of inflammation have been postulated previously by others in the context of many different diseases, however, whether these biomarkers are clinically validated or have diagnostic value remains to be clarified. Here, we describe an approach to predict and verify candidate biomarkers that can allow assessment of local inflammation in the context of metabolic imbalance as early indicators of chronic low-grade inflammation.

A workflow was created for screening of large databases (GO database and Human Protein Atlas) to determine the overlap between liver and adipose tissue-specific genes and inflammation-related genes. The overlapping genes/proteins were then selected based on their previous utilization as circulating biomarker in human clinical studies as indicated in the Clarivate Analytics biomarker database. Verification with murine gene expression data and human biomarker literature on tissue-derived low-grade inflammation indicated the feasibility of the workflow, represented by in a list of 18 biomarkers, of which 16 candidate plasma biomarkers were correlated with hepatic inflammation and 2 candidate biomarkers with adipose tissue inflammation. It must be noted that currently applied biomarkers for chronic low grade inflammation [e.g., Interleukin 6, adiponectin and soluble tumor necrosis factor-a receptor 2 (sTNFRII)] were not automatically selected in our verification procedure as they are not found to be tissue-specific and/or not expressed in mice as acute phase protein (C-reactive protein) and not fulfilling the selection criteria (e.g., differentially expressed as genes in inflamed tissue) as described in M&M.

**FIGURE 5:**
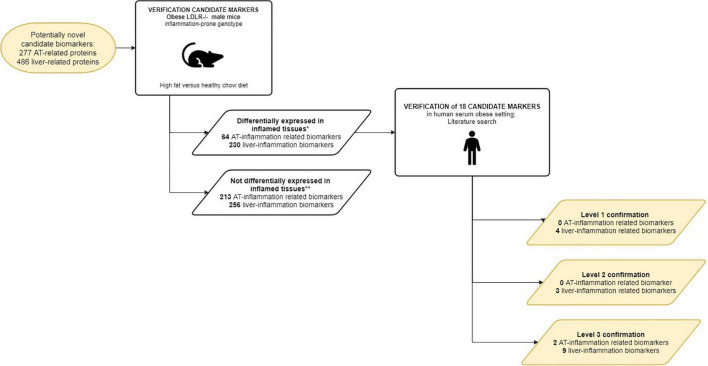
Workflow of the verification of the curated candidate biomarkers. Curated candidate biomarkers were checked for up/down regulated gene-expression in WAT and livers of obese mice. The curated candidate biomarkers were divided into 2 groups: markers differentially expressed in inflamed tissues; markers not differentially expressed in inflamed tissues. *Cut-off criteria described in M&M; ** Due to absence of murine analog of human candidate biomarker and/or lack of (differential) expression in murine tissues in RNAseq experiment.

Eleven out of 18 candidate biomarkers could be confirmed by human studies with the highest level of confirmation (Table 3). It should be noted that the number of verified candidate biomarkers was restricted to the genes that are also expressed in murine tissue (Figure 5) suggesting that the actual yield of the approach can be larger than described here.

The candidate biomarkers with the highest level of confirmation included extracellular matrix remodeling proteins, serine proteases (inhibitors), lipid metabolism related proteins, and mediators of immune activation (Table 4). Further study of the selected candidate biomarkers confirmed the selection procedure:

**TABLE 4:** Biological function of the 11 human candidate biomarkers with the highest level of confirmation (level 3).

**Biological function**	**Candidate biomarkers Correlated with hepatic inflammation**	**Candidate biomarkers Correlated with AT inflammation**
Remodeling extracellular matrix	–	Matrix metalloproteinase-9 (MMP-9)
Lipid metabolism	Leptin receptor (LEPR), Low Density Lipoprotein receptor (LDLR)	Resistin (RETN)
Mediators immune activation	Serum Amyloid A1 (SAA1), Haptoglobin (HP), Mannose Binding Lectin 2 (MBL2), Interleukin 18 binding protein (IL18BP)	–
Serine protease inhibitors	Alpha 1-antitrypsin (SERPINA1), Alpha-1-anti chymotrypsin (SERPINA3), Anti-thrombin (SERPINC1)	–

MMP-9 is related to remodeling of extracellular matrix processes and inflammation. MMP-9 regulates inflammatory processes by its proteolytic activity (Manicone and Mcguire, 2008) and circulating levels of MMP-9 are increased in obesity, metabolic syndrome (MetS) and cardiovascular disease (Hopps et al., 2016; Jaoude and Koh, 2016; Ritter et al., 2017).

Serine protease inhibitors (SERPINs) are key regulators of numerous biological pathways that are principally involved in regulation of the inflammatory cascades by enzyme activity modification, fibrinolysis, complement activation and kinin release. Alpha1-antitrypsin (a1AT) is produced in the liver and protects tissues from damage caused by proteolytic enzymes of inflammatory cells, especially neutrophil elastase (Janciauskiene et al., 2018). Similarly, alpha-1-anti chymotrypsin protects tissues from damage by inhibiting the activity of cathepsin G that is found in neutrophils and chymases found in mast cells (Kalsheker, 1996). Antithrombin, for example modulates inflammatory responses not only by inhibiting thrombin and other clotting factors that induce cytokine activity and leukocyte endothelial cell interaction, but also by coagulation independent effects, including direct interaction with cellular mediators of inflammation (Levy et al., 2016). Together, these findings confirm the selection procedure by which these 3 members of the SERPIN family were selected as candidate biomarkers correlated with hepatic inflammation.

Three of the highest level candidate biomarkers that were negatively associated with inflammation, were involved in lipid metabolism, *viz.* LDLR, LEPR, and RETN coding, respectively, for low density lipoprotein receptor, leptin receptor and resistin. Resistin is a pro-inflammatory adipokine associated with insuline resistance and obesity (Bokarewa et al., 2005; Su et al., 2019). LDLR and LEPR are in part related to triglyceride and cholesterol metabolism in the liver. The hepatic LDL receptor plays an important role in the clearance of cholesterol-rich LDL lipoproteins from circulation to the liver (Brown and Goldstein, 1983). We have shown, in animal studies that increased clearance of these cholesterol-rich LDL particles contribute to increased hepatic inflammatory response (Kleemann et al., 2007). Leptin is a circulating adipokine derived from adipose tissue regulating food intake, insulin action and modulation of the immune system. These biological processes are initiated by the leptin receptor upon interaction with circulating leptin. In parallel, Leptin receptor is known to activate Janus kinase-STAT3 pathway (Ikejima et al., 2004) which contributes to an inflammatory response (Kubler, 2014).

Four of the selected candidate biomarkers are involved in direct activation of the immune pathways. Haptoglobin and SAA1 are well-known acute phase proteins produced by hepatocytes. Their plasma levels are increased rapidly in subjects following an inflammatory trigger. Our data shows their gene expression is positively correlated with inflammation in liver. Mannose-binding lectin 2 plays an important role in the innate, non-specific immunity, by activating complement resulting among others in cell lysis, phagocytosis, and inflammation (Beltrame et al., 2015). IL-18 Binding Protein was positively associated with hepatic inflammation, which could be confirmed by literature. IL18BP binds and neutralizes the pro-inflammatory cytokine IL-18, thereby inhibiting IL18-induced IFNgamma production (Dinarello et al., 2013).

The approach described here is promising, but the verification of this approach also has its limitations. One important limitation is that samples from human studies with well-characterized tissue inflammation and corresponding serum/plasma samples were not available to validate the biomarkers. Alternatively we used published human studies to validate the presence of predicted biomarkers in serum/plasma, and used animal data to link biomarkers to tissue inflammation. TAlso, it is known that literature is biased toward studies in which effects were observed and raw datasets are often not made available thereby limiting direct validation possibilities. As an alternative, we have used mouse tissue and plasma samples from preclinical studies, which were limited by the use of one particular mouse strain, the use of one particular diet instead of multiple different liver and adipose tissue evoking diets, and the use of one gender (male mice). Another important consideration may be that biomarkers could be synthesized before inflammation in liver or adipose tissue becomes manifest. Therefore it could be possible that biomarkers do not always correlate with cellular inflammatory features. Therefore, future studies for the identification of early markers of tissue inflammation would require dedicated studies (preferably time resolved).

One important issue is the validity to translate the experimental murine data to humans (Perlman, 2016), given the known difference in metabolism of mice and humans. It is known that the metabolic rate of mice version human is closely correlated with size, thus a 30 g mouse has a specific metabolic rate (metabolic rate per gram of tissue) roughly seven times that of a 70-kg human (Kleiber, 1961; Schmidt-Nielsen and Knut, 1984). Mice have relatively higher amounts of metabolically active tissues, such as liver and kidney and differ in the mitochondrial density and metabolic rate and also in the fatty acid composition of their membrane phospholipids [higher contents of polyunsaturated fatty acid docosahexaenoic acid (Hulbert, 2008)]. Despite these differences in metabolism between mice and humans, we have shown previously that the key molecular inflammatory responses in obese Ldlr−/−.Leiden mice and humans are similar (Morrison et al., 2018): the hepatic inflammatory response in NASH patients (non-alcohol steatohepatitis; due to an increased metabolic load) has large similarities to the obese Ldlr−/−.Leiden mice fed with a HFD demonstrated by the activation of the majority of identical inflammatory processes and master regulators (e.g., TNF, CSF2, TGFB1) (Morrison et al., 2018). Moreover, the candidate biomarkers that were verified in the obese Ldlr−/−.Leiden mice were further evaluated for their confirmed use as hepatic or adipose inflammation in human, albeit in a different disease context. Therefore we believe it is valid to translate the identified biomarkers to the human situation, although they still require validation in a human study.

Our study shows that by combining prior knowledge from multiple studies we were able to select and verify a set of *in silico* predicted biomarkers for liver and adipose tissue-derived inflammation. These biomarkers may be of great help to form a starting point to clarify tissue-specific inflammatory mechanisms. Tissue-specific chronic low-grade inflammation is an important underlying contributor to complications of metabolic disorders. To support therapeutic approaches to these complications, it is crucial to gain a deeper understanding of the inflammatory dynamics and to monitor them on the individual level. A hugh advantage of our approach is that our workflow is for a substantial part automated (database searches) which enables the screening of enormous amounts of data, thereby saving time and resources.

As one of the critical selection criteria of our approach was the selection based on the ability to detect these candidate markers as proteins in human plasma. This made it plausible that these candidate biomarkers can easily be implemented in future human studies to monitor or predict liver- and adipose tissue inflammation. This will become of great value to study future development of therapeutic/lifestyle interventions to combat metabolic inflammatory complications. Our next step will be to validate these selected candidate biomarkers in a human nutritional study as proof-of-concept of our approach.

## Conclusion

We describe a promising Systems Biology approach that predicts tissue-derived, blood-based biomarkers reflecting liver- and adipose tissue inflammation that may be of great use to gain more mechanistic knowledge on tissue-specific inflammation and to monitor or predict the efficacy of interventions in metabolic inflammatory conditions.

## Data Availability Statement

The original contributions presented in the study are included in the article/Supplementary Material, further inquiries can be directed to the corresponding author.

## Ethics Statement

The animal study was reviewed and approved by Animal experimental commission TNO, approval reference number TNO-312.

## Author Contributions

JB, WB, SW, and LV contributed to the conception and design of the study. RD and MC wrote scripts for literature database searches and biomarker selection. AH and RK contributed to the animal experiment. JB, WB, and LV wrote the manuscript. All authors contributed to manuscript revision, read, and approved the submitted version.

## Conflict of Interest

The authors declare that the research was conducted in the absence of any commercial or financial relationships that could be construed as a potential conflict of interest.

## Publisher’s Note

All claims expressed in this article are solely those of the authors and do not necessarily represent those of their affiliated organizations, or those of the publisher, the editors and the reviewers. Any product that may be evaluated in this article, or claim that may be made by its manufacturer, is not guaranteed or endorsed by the publisher.

## Footnotes

1
www.geneontology.org

2
www.proteinatlas.org

3
https://www.cortellis.com/intelligence/
