# Supplementary material for: Mechanism-Based Biomarker Prediction for Low-Grade Inflammation in Liver and Adipose Tissue
Source: Front Physiol. 2021 Nov 10;12:703370. doi: 10.3389/fphys.2021.703370 (PMC8631400; doi:10.3389/fphys.2021.703370)
Supplement: Supplementary file 1 [file Table_1.pdf]

Table S1 - overview of adipose ontology search result and the selection of parent gene ontologies

included = ontology included
is part_of = ontology not included because it is part of another included parent ontology
not within scope = not included because not specific to adipose tissue in humans

Selection	GO ID	GO term
included	GO:0060612	adipose tissue development
is part_of	GO:1904178	negative regulation of adipose tissue development
is part_of	GO:1904179	positive regulation of adipose tissue development
is part_of	GO:1904177	regulation of adipose tissue development
included	GO:1904606	fat cell apoptotic process
is part_of	GO:1904649	regulation of fat cell apoptotic process
is part_of	GO:1904651	positive regulation of fat cell apoptotic process
is part_of	GO:1904650	negative regulation of fat cell apoptotic process
not within scope	GO:0061285	mesonephric capsule development
not within scope	GO:0061286	mesonephric capsule morphogenesis
not within scope	GO:0061287	mesonephric capsule formation
is part_of	GO:0060613	fat pad development
is part_of	GO:0060611	mammary gland fat development
included	GO:0070162	adiponectin secretion
included	GO:0045444	fat cell differentiation
is part_of	GO:0070347	regulation of brown fat cell proliferation
is part_of	GO:0070346	positive regulation of fat cell proliferation
is part_of	GO:0070349	positive regulation of brown fat cell proliferation
is part_of	GO:0070348	negative regulation of brown fat cell proliferation
is part_of	GO:0070350	regulation of white fat cell proliferation
is part_of	GO:0070352	positive regulation of white fat cell proliferation
is part_of	GO:0070351	negative regulation of white fat cell proliferation
included	GO:0070341	fat cell proliferation
is part_of	GO:0070343	white fat cell proliferation
is part_of	GO:0070342	brown fat cell proliferation
is part_of	GO:0070345	negative regulation of fat cell proliferation
is part_of	GO:0070344	regulation of fat cell proliferation
not within scope	GO:0072213	metanephric capsule development
not within scope	GO:0072265	metanephric capsule morphogenesis
not within scope	GO:0072266	metanephric capsule formation
not within scope	GO:0072128	renal capsule morphogenesis
not within scope	GO:0072129	renal capsule formation
not within scope	GO:0072127	renal capsule development
is part_of	GO:0033210	leptin-mediated signaling pathway
is part_of	GO:0044320	cellular response to leptin stimulus
included	GO:0044321	response to leptin
is part_of	GO:0090335	regulation of brown fat cell differentiation
is part_of	GO:0090336	positive regulation of brown fat cell differentiation
is part_of	GO:0050873	brown fat cell differentiation
is part_of	GO:0050872	white fat cell differentiation
is part_of	GO:0060642	white fat cell differentiation involved in mammary gland fat development
is part_of	GO:0045599	negative regulation of fat cell differentiation
is part_of	GO:0045598	regulation of fat cell differentiation

is part_of	GO:0045600	positive regulation of fat cell differentiation
is part_of	GO:1903444	negative regulation of brown fat cell differentiation
included	GO:0055100	adiponectin binding
included	GO:0005901	caveola
