# Supplementary material for: Mechanism-Based Biomarker Prediction for Low-Grade Inflammation in Liver and Adipose Tissue
Source: Front Physiol. 2021 Nov 10;12:703370. doi: 10.3389/fphys.2021.703370 (PMC8631400; doi:10.3389/fphys.2021.703370)
Supplement: Supplementary file 2 [file Table_2.pdf]

Table S2 - overview of hepatic ontology search result and the selection of parent gene ontologies

included = ontology included
is part_of = ontology not included because it is part of another included parent ontology
not within scope = not included because not specific to adipose tissue in humans

**Selection**	**GO ID**	**GO term**
is part_of	GO:0060344	liver trabecula formation
is part_of	GO:0097421	liver regeneration
not within scope	GO:1990402	embryonic liver development
is part_of	GO:0071910	determination of liver left/right asymmetry
is part_of	GO:0072575	epithelial cell proliferation involved in liver morphogenesis
is part_of	GO:0072576	liver morphogenesis
included	GO:0001889	liver development
not within scope	GO:0015234	thiamine transmembrane transporter activity
not within scope	GO:0015127	bilirubin transmembrane transporter activity
is part_of	GO:0090320	regulation of chylomicron remnant clearance
is part_of	GO:0090321	positive regulation of chylomicron remnant clearance
not within scope	GO:0051413	response to cortisone
included	GO:0036333	hepatocyte homeostasis
not within scope	GO:0036378	calcitriol biosynthetic process from calciol
not within scope	GO:0061011	hepatic duct development
not within scope	GO:0061009	common bile duct development
not within scope	GO:2001069	glycogen binding
not within scope	GO:0014831	gastro-intestinal system smooth muscle contraction
is part_of	GO:0035622	intrahepatic bile duct development
not within scope	GO:0045122	aflatoxin biosynthetic process
is part_of	GO:0070365	hepatocyte differentiation
is part_of	GO:0070367	negative regulation of hepatocyte differentiation
is part_of	GO:0070366	regulation of hepatocyte differentiation
is part_of	GO:0070368	positive regulation of hepatocyte differentiation
not within scope	GO:0030975	thiamine binding
not within scope	GO:0048175	hepatocyte growth factor biosynthetic process
not within scope	GO:0009228	thiamine biosynthetic process
not within scope	GO:0009230	thiamine catabolic process
not within scope	GO:0034363	intermediate-density lipoprotein particle
not within scope	GO:0034362	low-density lipoprotein particle
not within scope	GO:0034361	very-low-density lipoprotein particle
not within scope	GO:0034360	chylomicron remnant
not within scope	GO:0034364	high-density lipoprotein particle
included	GO:0034382	chylomicron remnant clearance
included	GO:0034379	very-low-density lipoprotein particle assembly
not within scope	GO:0006772	thiamine metabolic process
not within scope	GO:0004806	triglyceride lipase activity
not within scope	GO:0097330	response to 5-fluoro-2'-deoxyuridine
included	GO:0097284	hepatocyte apoptotic process
not within scope	GO:0043639	benzoate catabolic process
not within scope	GO:0043691	reverse cholesterol transport
not within scope	GO:0018952	parathion metabolic process
not within scope	GO:0018874	benzoate metabolic process

not within scope	GO:0042199	cyanuric acid metabolic process
included	GO:1990828	hepatocyte dedifferentiation
included	GO:1990922	hepatic stellate cell proliferation
not within scope	GO:0003046	regulation of systemic arterial blood pressure by stress relaxation
not within scope	GO:0002251	organ or tissue specific immune response
included	GO:0002194	hepatocyte cell migration
not within scope	GO:0002542	Factor XII activation
not within scope	GO:0002337	B-1a B cell differentiation
included	GO:0002384	hepatic immune response
not within scope	GO:0071388	cellular response to cortisone stimulus
not within scope	GO:0046222	aflatoxin metabolic process
not within scope	GO:0046223	aflatoxin catabolic process
not within scope	GO:0007503	fat body development
not within scope	GO:0071934	thiamine transmembrane transport
is part_of	GO:0072574	hepatocyte proliferation
not within scope	GO:0015888	thiamine transport
not within scope	GO:0019339	parathion catabolic process
is part_of	GO:1904898	negative regulation of hepatic stellate cell proliferation
is part_of	GO:1904897	regulation of hepatic stellate cell proliferation
is part_of	GO:1904899	positive regulation of hepatic stellate cell proliferation
included	GO:0061521	hepatic stellate cell differentiation
included	GO:0061872	hepatic stellate cell contraction
is part_of	GO:0061873	regulation of hepatic stellate cell contraction
is part_of	GO:0061874	positive regulation of hepatic stellate cell contraction
is part_of	GO:0061875	negative regulation of hepatic stellate cell contraction
is part_of	GO:0061870	positive regulation of hepatic stellate cell migration
is part_of	GO:0061871	negative regulation of hepatic stellate cell migration
is part_of	GO:0061869	regulation of hepatic stellate cell migration
included	GO:0061868	hepatic stellate cell migration
not within scope	GO:0061007	hepaticobiliary system process
not within scope	GO:0061008	hepaticobiliary system development
included	GO:0035733	hepatic stellate cell activation
is part_of	GO:2000491	positive regulation of hepatic stellate cell activation
is part_of	GO:2000490	negative regulation of hepatic stellate cell activation
is part_of	GO:2000489	regulation of hepatic stellate cell activation
not within scope	GO:0010901	regulation of very-low-density lipoprotein particle remodeling
not within scope	GO:0010903	negative regulation of very-low-density lipoprotein particle remodeling
not within scope	GO:0010902	positive regulation of very-low-density lipoprotein particle remodeling
not within scope	GO:0034369	plasma lipoprotein particle remodeling
not within scope	GO:0034374	low-density lipoprotein particle remodeling
not within scope	GO:0034372	very-low-density lipoprotein particle remodeling
not within scope	GO:0034375	high-density lipoprotein particle remodeling
not within scope	GO:0004454	ketohexokinase activity
is part_of	GO:2000346	negative regulation of hepatocyte proliferation
is part_of	GO:2000345	regulation of hepatocyte proliferation
is part_of	GO:2000347	positive regulation of hepatocyte proliferation
not within scope	GO:0036458	hepatocyte growth factor binding
not within scope	GO:2001113	negative regulation of cellular response to hepatocyte growth factor stimulus
not within scope	GO:2001114	positive regulation of cellular response to hepatocyte growth factor stimulus
not within scope	GO:2001112	regulation of cellular response to hepatocyte growth factor stimulus

not within scope	GO:0035729	cellular response to hepatocyte growth factor stimulus
not within scope	GO:0035728	response to hepatocyte growth factor
not within scope	GO:0048178	negative regulation of hepatocyte growth factor biosynthetic process
not within scope	GO:0048177	positive regulation of hepatocyte growth factor biosynthetic process
not within scope	GO:0048176	regulation of hepatocyte growth factor biosynthetic process
not within scope	GO:0048012	hepatocyte growth factor receptor signaling pathway
not within scope	GO:0005008	hepatocyte growth factor-activated receptor activity
not within scope	GO:0005171	hepatocyte growth factor receptor binding
not within scope	GO:1902202	regulation of hepatocyte growth factor receptor signaling pathway
not within scope	GO:1902203	negative regulation of hepatocyte growth factor receptor signaling pathway
not within scope	GO:1902204	positive regulation of hepatocyte growth factor receptor signaling pathway
not within scope	GO:0032726	positive regulation of hepatocyte growth factor production
not within scope	GO:0032646	regulation of hepatocyte growth factor production
not within scope	GO:0032686	negative regulation of hepatocyte growth factor production
not within scope	GO:0032605	hepatocyte growth factor production
is part_of	GO:1903944	negative regulation of hepatocyte apoptotic process
is part_of	GO:1903945	positive regulation of hepatocyte apoptotic process
is part_of	GO:1903943	regulation of hepatocyte apoptotic process
is part_of	GO:0015126	canalicular bile acid transmembrane transporter activity
included	GO:0033675	pericanalicular vesicle
is part_of	GO:0061017	hepatoblast differentiation
included	GO:0015722	canalicular bile acid transport
